# Transcriptomic Exploration of Muscle Development and Fat Deposition Trait Diversity in Selected Indian Sheep Breeds: Implications for Meat Quality and Yield

**DOI:** 10.3390/ani16030452

**Published:** 2026-02-01

**Authors:** Navya Pothireddy, Mangalathu Rajan Vishnuraj, Kappala Vijaya Rachel, Peddapuram Baswa Reddy, Prashantha Chowdadenahalli Nagaraja, Ajay Ganesan, Shiva Shankar Kanneboyina, Krishnachaithanya Indiradevi, Sukhadeo Baliram Barbuddhe

**Affiliations:** 1 Department of Life Science (Biochemistry), GITAM University, Rushikonda, Visakhapatnam 530045, Andhra Pradesh, India; npothire2@gitam.in; 2 ICAR-National Meat Research Institute, Chengicherla, Hyderabad 500092, Telangana, India; baswareddy@gmail.com (P.B.R.); ajaybiotechbdu@gmail.com (A.G.); shivakanneboyina@gmail.com (S.S.K.); krishnachaithanya03i@gmail.com (K.I.); barbuddhesb@gmail.com (S.B.B.); 3 School of Applied Sciences, REVA University, Bangalore 560064, Karnataka, India; prashantha.cn@reva.edu.in

**Keywords:** transcriptomics of meat quality, Nellore and Deccani sheep breeds, muscle growth, intramuscular fat

## Abstract

Sheep’s meat production and quality are influenced by genetic and physiological factors that affect muscle development, growth, and fat deposition metabolism. However, the breed-specific transcriptional landscapes driving these traits in Indian sheep breeds, especially in Nellore (meat-type) and Deccani (wool-meat type) breeds are remain unexplored. Therefore, this study aimed to investigate the differences in muscle growth and fat deposition between Nellore and Deccani breeds by integrating transcriptomic profiling, carcass characteristics, and histological analysis of longissimus dorsi muscle and liver tissues. Carcass assessment revealed higher Hot Carcass Weight (HCW), Cold Carcass Weight (CCW), Hot Carcass Yield (HCY) and Cold Carcass Yield (CCY), and larger myofibrillar cross-sectional area (*p* < 0.05), indicating enhanced musculature, which was observed in Nellore. Deccani showed elevated Intramuscular Fat (IMF) deposition (*p* < 0.05), indicating improved meat flavour/juiciness. Transcriptomic profiling revealed several Differentially Expressed Genes (DEGs) associated with meat quality and quantity traits. In Nellore, the genes WFIKKN2, FGFRL1, FKBP4, and IRF1 were upregulated, while the gene TAS1R2 was downregulated, leading to enhanced muscle development, superior carcass traits, thermotolerance, and immunity. While Deccani showed higher expression of lipid metabolism genes PLA2G4F, ACSL1, ACOX1, CPT1A, and PLIN1, which are linked to higher IMF content. Functional enrichment analysis revealed 46 significantly enriched GO terms for the DEGs (*p* < 0.05), including oxidoreductase activity, muscle development, etc. These outcomes demonstrate novel genetic markers and key biological insights into the regulation of muscle development, thermotolerance, immunity, and IMF for future validation in Indian sheep breeds.

## Simple Summary

This study aimed to explore the muscle development and fat deposition trait diversity in Nellore (meat-type) and Deccani (wool-meat type) sheep breeds of India by integrating transcriptomics profiling, carcass characteristics, and histological analysis of longissimus dorsi muscle and liver tissues. Differential gene expression analysis revealed the genes that are associated with muscle development and fat deposition, while the functional enrichment analysis unveiled the underlying molecular mechanisms involved in lipid storage, triglyceride synthesis, muscle development, mitochondrial oxidation, etc. As the first comparative investigation of transcriptomics profiles, carcass characteristics and histological analysis of myofibrillar cross-sectional area between Nellore and Deccani breeds of India, it not only enhances our understanding of genotype–phenotype relationships in the selected Indian sheep but also lays the foundation for genomic selection strategies aimed at improving meat production and quality.

## 1. Introduction

Meat is one of the most important sources of nutrients in the human diet, playing a crucial role in global food security and nutrition [1]. In contrast to other red meats like beef and pork, mutton is high in unsaturated fatty acids, vitamins (B2 and B12) and minerals (calcium, magnesium, potassium, selenium); Further, its digestibility in humans is high (90%) [2]. World sheep meat production has reached 16.4 million tons in 2021, with an average annual growth rate of 1.8% and is anticipated to reach 19 million tons by 2034 (baseline year 2025) [3]. The total sheep meat intake has grown rapidly in recent years and is anticipated to rise by 16% over the period 2025–2034. During the period 2019–2021, India is the second-largest consumer of mutton and is poised to increase by 14% by 2031 [3].

India is among the few selected nations that have made significant contributions to the global livestock gene pool. Currently, India is home to 46 indigenous sheep breeds, which are well-adapted to diverse agro-climatic conditions and are registered by the Indian Council of Agricultural Research—National Bureau of Animal Genetic Resources (ICAR-NBAGR) [4]. These sheep breeds are crucial for meeting the consumer’s ever-growing demand for ethically sourced, nutritious, and high-quality meat [5,6]. Out of the 46 indigenous sheep breeds, the Nellore breed has the largest share, at 20%, followed by the breeds Deccani, Marwari, Bellari, Jaisalmeri, and Mecheri. Andhra Pradesh and Telangana States from the Deccan plateau region are leading states in sheep rearing, accounting for 23.7% and 25.7% of the nation’s total sheep population, respectively [7]. The Nellore sheep, predominantly found in these states, are the preferred animals for commercial meat production because of their lean meat and rapid growth potential. On the other hand, Deccani sheep, found in the Deccan Plateau areas of Telangana, Karnataka, and Maharashtra, are dual-purpose, producing both meat and wool [8,9]. Breed’s growth rate, organoleptic qualities, and overall nutritional value of meat are determined by the environmental conditions, genetic attributes and nutrition [10]. Despite their importance in local agricultural systems, their genetic basis for meat production and quality performance is unexplored.

The quality and production of meat are multifactorial systems that can be driven by several biological factors, including gene expression, metabolism, and postmortem physiology, all of which function together through intricate mechanisms [11]. The emergence of RNA-sequencing (RNA-Seq) technology has significantly enhanced our ability to explore the complex genetic and physiological relationships governing meat production and quality in livestock [12]. In various livestock species, including cattle [13], goats [14], pigs [15], sheep [16], and chickens [16], this approach has made an invaluable contribution to identifying Differentially Expressed Genes (DEGs) linked to muscle growth, development, and meat quality. Such findings emphasise the genomic screening strength in identifying the candidate markers which can later be validated for functional Single Nucleotide Polymorphisms (SNPs). These validated markers support the Marker-Assisted Selection (MAS) programmes, which aims to improve the meat production and quality attributes in livestock [17]. Although these genetic breakthroughs provide valuable insights, further investigations are still needed to thoroughly comprehend the genotype-phenotype connections among the Deccani and Nellore sheep breeds. Given their importance in regional farming practices, it is necessary to investigate their genetic background for meat production and quality.

In the present study, we have analysed the longissimus dorsi muscle and liver tissues of Deccani and Nellore sheep breeds through comparative transcriptomic profiling. Studies are carried out to observe the transcriptomic differences across the breeds and tissue comparisons, to understand the tissue-specific regulatory patterns influencing IMF fat deposition. This research aims to identify the key regulatory genes for future genetic improvement initiatives focused on enhancing meat production in terms of both quantity and quality in sheep.

## 2. Materials and Methods

### 2.1. Experimental Animals and Sample Collection

The present study was conducted at the ICAR-National Meat Research Institute (ICAR-NMRI), Hyderabad, India. Eight healthy non-castrated males from the Deccan Plateau region in India were selected and divided into two groups: one comprised four Deccani sheep and the other four sheep from the Nellore breed. All the animals, aged between 9 and 12 months, were chosen based on yield-related traits and were reared under a uniform extensive farming management system typical of the region, utilising free-range grazing only on the same pasture to ensure identical feeding management and environmental exposure. Routine health management practices, including deworming and vaccination were followed according to veterinary doctor recommendations. The study was conducted in 2024, spanning a single season (March to June), to maintain consistent ambient environmental conditions. The animals were purchased from an authentic source, and the transport distance is approximately 1.2 kilometres. All animals underwent a 24 h fasting period with ad libitum access to water to reduce preslaughter stress and ensure standardisation in pre-slaughter metabolic conditions [18]. The live body weights were recorded prior to slaughter, and the sheep were then scientifically slaughtered by the electrical stunning method at the ICAR-NMRI (FSSAI-approved slaughterhouse) to minimise the stress during slaughter, in accordance with animal welfare guidelines. Critical to maintaining experimental control, the four animals (two from each breed) used for transcriptomics analysis were slaughtered and sampled simultaneously on the same day. Post-mortem, the longissimus dorsi muscle and liver tissue samples were rapidly excised and preserved in RNAlater™ solution to stabilise and protect RNA integrity for transcriptomic analysis. The carcasses were subsequently chilled in a cold storage room maintained at 4 °C for 24 h. For histological evaluations, a portion of the collected tissues was fixed in Neutral Buffer Formalin, and the other portion was embedded in Optimal Cutting Temperature (OCT) media and stored at ultra-low temperatures until further processing.

### 2.2. Histological Analysis

#### 2.2.1. Oil Red O Staining and Lipid Quantification

Frozen tissue samples were cryosectioned at a thickness of 10 μm using a Leica CM3050S cryostat (Leica Microsystems GmbH, Wetzlar, Germany), and the sections were carefully mounted onto clean glass slides. Tissue sections were fixed in 10% neutral buffered formalin for 15 min, followed by a rinse in distilled water and brief immersion in 60% isopropyl alcohol to prepare the tissue for staining. Oil Red O staining was performed using a freshly prepared working solution, and the sections were incubated for 20 min. After staining, slides were rinsed sequentially with 60% isopropyl alcohol and distilled water to remove excess dye. Counterstaining was conducted with acidified Lillie-Mayer hematoxylin, and bluing was achieved using 0.3% sodium borate. Finally, the stained sections were mounted with glycerin jelly and examined under a light microscope. For quantitative analysis, images were captured at 200× magnification from six randomly selected fields (*n* = 6) per tissue section [19,20]. The lipid droplets, indicated by red staining, were analysed using ImageJ^®^ software (v1.54a). A uniform red colour threshold was applied across all images to ensure consistency in quantification. The percentage of lipid droplets was calculated using the formula:

Lipid Droplets Percentage = (Red Droplet Area)/(Total Tissue Area) × 100

#### 2.2.2. Measurement of Myofibrillar Cross-Sectional Area & Sarcomere Length

Myofibrillar cross-sectional area and sarcomere were measured using tissue sections stained with Hematoxylin and Eosin (H&E) stain. The images were captured at 200× magnification for myofibrillar cross-sectional area and at 400× magnification for sarcomere length [21]. For each sample, 4–5 randomly selected myofibrils and sarcomeres from five consecutive sections per muscle sample were measured using Zeiss Zen 3.9 image analysis software (Oberkochen, Germany).

#### 2.2.3. Quantification of Intramuscular Collagen

Intramuscular collagen content was quantified using tissue sections stained with 0.1% picrosirius red. Twenty random fields of view were selected for each muscle sample across four consecutive sections. Images were captured under consistent microscope settings (at 10×, 40× and 100× objective lens magnification) and analysed using ImageJ^®^ software. Collagen was quantified by setting a grey value threshold between 20 and 255. The mean percentage of collagen-covered area and standard error were used to express the results [21].

### 2.3. Transcriptomics Analysis

#### 2.3.1. RNA Extraction and Sequencing

Total RNA was extracted from the longissimus dorsi muscle and liver tissues using the TRIzol™ reagent (Invitrogen, Carlsbad, CA, USA) according to the manufacturer’s protocol. The concentration and purity of the isolated RNA were assessed using a NanoPhotometer^®^ (IMPLEN, Munich, Germany), ensuring optimal absorbance ratios (A260/A280) for downstream applications. The RNA integrity of each sample was evaluated using the QIAxcel Advanced capillary electrophoresis system (Qiagen, Hilden, Germany). Only RNA samples with an RNA Integrity Number (RIN) between 5.0 and 7.0 were selected for library preparation, indicating acceptable RNA quality for transcriptomic studies. For transcriptome analysis, RNA-Seq libraries were prepared using the NEBNext^®^ Ultra™ RNA Library Prep Kit for Illumina^®^ platforms (New England Biolabs, Ipswich, MA, USA), following the manufacturer’s guidelines. The resulting libraries were sequenced using the Illumina NovaSeq 6000 platform (Illumina, Inc., San Diego, CA, USA), configured for paired-end sequencing (2 × 150 bp) to ensure high coverage and depth suitable for differential gene expression analysis [22].

#### 2.3.2. RNA Seq Data Analysis

To examine differential gene expression profiles in Deccani and Nellore sheep, RNA-seq data obtained from longissimus dorsi muscle and liver tissues were subjected to a comprehensive bioinformatics pipeline. This included data preprocessing, alignment, quantification, differential gene expression analysis, and functional annotation to elucidate breed-specific physiological characteristics, such as meat quality, lipid metabolism, stress response, and growth performance. Bioinformatics approaches were used to demultiplex the sequence data, filter the sequence duplicates, and store the datasets in FASTq file format. The raw sequence data is used for quality analysis, trimming and mapping. The raw data quality was checked using FastQC (v0.11.9) [23] and MultiQC [24] software (v1.33). The data was checked for base call quality distribution, percentage of bases above Q30, percentage of GC content, and sequencing adapter contamination. The raw sequence reads were processed to remove adapter sequences and low-quality bases using fastp v0.12.4 [25] with default parameters. Reads shorter than 50 bp were removed from further analysis. From the quality-trimmed data, rRNA reads were removed using the BBMAP v38.18 [26] bbduk algorithm. Post-trimming, another round of FastQC verified the effectiveness of the cleaning step, improving sequence reliability for alignment. Clean reads were aligned to the Ovis aries [Oar_v3.1 (GCA_000298735.1)] reference genome using HISAT2 (v2.2.1), a fast and sensitive alignment tool designed for spliced reads, especially important in transcriptome studies where exon-intron boundaries need to be correctly resolved. The alignment step yielded sorted and indexed BAM files using SAMtools (v1.10), providing a structured format for downstream analysis. Gene expression was quantified using FeatureCounts from the Subread package (v2.1.1), which assigned aligned reads to specific gene annotations. Gene expression levels were calculated using the FPKM method (Fragments Per Kilobase of transcript per Million mapped reads). This step generated a count matrix reflecting the expression levels of thousands of genes across all tissue samples and breeds, forming the basis for identifying transcriptional differences related to breed-specific traits. This preprocessing step was critical to ensure accurate and reproducible mapping, particularly when working with large RNA-seq datasets from complex tissues like muscle and liver, which are metabolically active and exhibit high transcriptomic diversity [23,27,28].

#### 2.3.3. Differential Gene Expression Analysis

To identify Differentially Expressed Genes (DEGs) between Deccani and Nellore breeds, DESeq2 (v1.30.1) was used. Using the median-of-ratios method, this tool normalised the gene count data and applied a negative binomial generalised linear model to determine expression changes. Genes with an adjusted *p*-value (FDR) < 0.05, and log-fold change ≥ 2 were considered statistically significant. Principal Component Analysis (PCA) and rlog transformation were used for exploratory data visualisation and quality assessment. DEGs were clustered by unsupervised hierarchical clustering based on comparing Deccani Muscle vs. Nellore Muscle, Deccani Liver vs. Nellore Liver, Deccani Muscle vs. Deccani Liver, and Nellore Liver vs. Nellore Muscle. A distance matrix is constructed based on calculating the distance between two samples. The samples can appear in the same cluster through clustering, and genes in the same cluster may have similar biological functions [24,25]. The UpSetR v1.4.0 R package was used to generate plots showing overlapping significant genes between conditions.

#### 2.3.4. Functional Enrichment of DEGs

DEGs were analysed based on DESeq2, and the upregulated and downregulated genes were functionally annotated and subjected to enrichment analysis using Enrichr (v2.42.0) and DAVID Functional Annotation, a widely used gene set enrichment tool that integrates various genomic databases [26]. Genes were classified into three major Gene Ontology (GO) categories: Biological Process (BP), Molecular Function (MF), and Cellular Component (CC), to understand their potential biological roles and localization. Enrichment significance was assessed using the hypergeometric distribution test, allowing the identification of GO terms significantly overrepresented among DEGs. This analysis enabled the prioritisation of key genes for further validation based on their biological relevance to the studied traits. Additionally, Kyoto Encyclopaedia of Genes and Genomes (KEGG) pathway enrichment analysis was performed to identify biologically meaningful pathways associated with DEGs [29,30,31]. Graphical representations, including volcano plots, heatmaps, and boxplots, were created using ggplot2 (v3.3.5) and heatmap 2 in R (v4.5.0) to visualise DEG patterns across tissue types and breeds.

### 2.4. Statistical Analysis

The unpaired two-tailed Student’s *t*-test (parametric) was performed for the Histological analysis and Carcass characteristics using GraphPad Prism (v5.01).

## 3. Results

### 3.1. Carcass Characteristics

The comparative analysis of the carcass characteristics between Nellore and Deccani sheep breeds is reported in Table 1. The Nellore breed had a significantly higher slaughter body weight (23.96 ± 0.89 kg) than the Deccani breed (19.50 ± 0.96 kg), indicating better growth performance with a *p*-value of 0.0146. Similarly, Hot Carcass Weight (HCW) and Cold Carcass Weight (CCW) were significantly greater in Nellore sheep than in Deccani sheep, with *p*-values of 0.0032 and 0.0048, respectively. In terms of yield, Hot Carcass Yield (HCY) and Cold Carcass Yield (CCY) were also significantly higher in the Nellore breed than in the Deccani breed, with *p*-values of 0.0215 and 0.0330, respectively. These results suggest that Nellore sheep are more efficient in converting live weight into usable meat. On the other hand, cooling loss, which measures the percentage of weight lost during the chilling process, did not differ significantly (*p* = 0.4010) between the two breeds, indicating similar moisture loss during refrigeration.

### 3.2. Muscle Histology and Microstructural Characteristics

**Figure 1:**
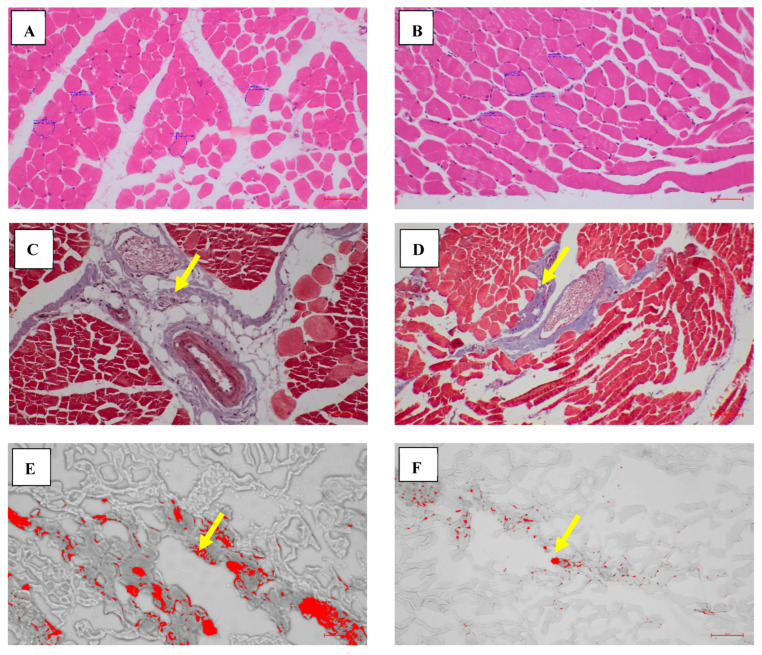
Longissimus dorsi sections of Nellore and Deccani sheep breeds. (**A**,**B**) Myofibrillar cross-sectional area (in 50 μM scale) in Deccani & Nellore breed, respectively, using H&E staining; (**C**,**D**) Collagen Content (in 100 μM scale) (Arrow mark showing Reddish blue coloured collagen fibres) in Deccani & Nellore breed, respectively, using picrosirius red staining; (**E**,**F**) IMF content [(in 50 μM scale)—(Arrow mark showing Red coloured IMF droplets)] in Deccani & Nellore breed, respectively, using Oil red O staining.

Histological analysis of the longissimus dorsi muscle demonstrated apparent differences in muscle fibre morphology and Intramuscular Fat (IMF) deposition between Nellore and Deccani sheep breeds (Figure 1).

**Figure 2:**
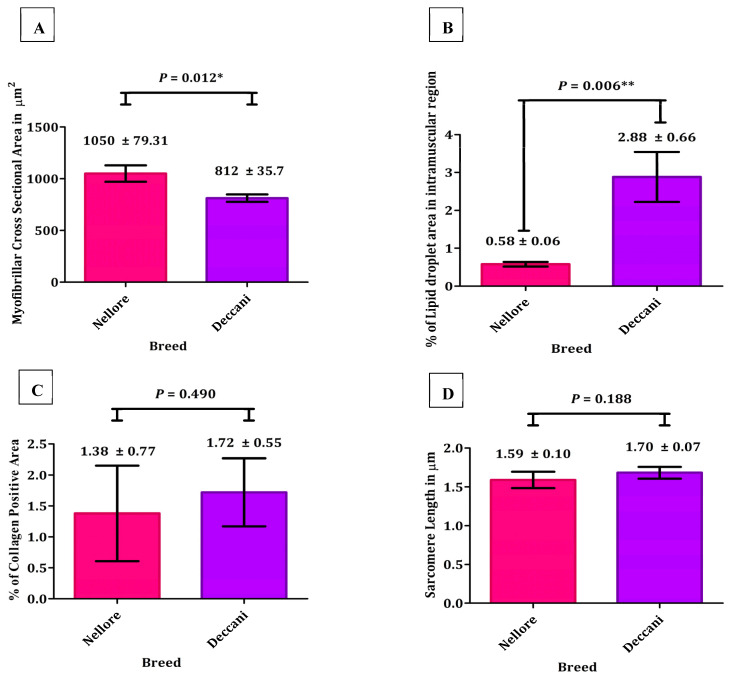
Structural and histological characteristics of muscle in Nellore and Deccani sheep breeds. (**A**) Myofibrillar cross-sectional area (μm^2^); (**B**) IMF content measured (as % of lipid droplet area); (**C**) Collagen content (%); (**D**) Sarcomere length in (μm). Where * indicates significant differences at *p* < 0.05 and ** indicates significant differences at *p* < 0.01.

The myofibrillar cross-sectional area was significantly larger in Nellore sheep (1050 ± 79.31 μm^2^) than in the Deccani sheep (812 ± 35.7 μm^2^) (Figure 2A; *p* = 0.012), indicating a greater muscle fibre hypertrophy in the Nellore breed. In contrast, the IMF content, expressed as the percentage of lipid droplet area within muscle tissue, was significantly higher in Deccani sheep (2.88 ± 0.66%) compared to Nellore (0.58 ± 0.06%) (Figure 2B; *p* = 0.006), suggesting a greater capacity for intramuscular lipid deposition in the Deccani breed. The Oil Red O staining findings were in agreement with the chemically determined IMF (AOAC 991.36) levels in the same animals. Furthermore, no significant differences were observed between breeds in collagen content (Nellore: 1.38 ± 0.77%; Deccani: 1.72 ± 0.55%) (Figure 2C; *p* = 0.490), although slight variations were noted. Similarly, sarcomere length was also not significantly different between the groups (Nellore 1.59 ± 0.10 μm; Deccani 1.70 ± 0.07 μm) (Figure 2D; *p* = 0.188).

### 3.3. Transcriptome Analysis of Muscle and Liver Samples of Deccani and Nellore Sheep Breeds

A total of 2 sets of Deccani muscle and Nellore muscle samples and two sets of Deccani liver and Nellore liver samples are used for cDNA library construction. A 2 × 150 bp NovoSeq 6000 sequencer (Illumina, Inc., San Diego, CA, USA) was used to generate raw sequence data. A total of 734.34912 raw paired-end reads (196.2 GB) were generated, with an average of 57.8, 59.3, 56.04, and 24.41 million reads obtained from muscle samples, 49.06, 29.20, 42.23, and 49.00 million reads obtained from liver samples, respectively (Supplementary Table S1). All the samples have passed the QC threshold (Q30 > 90%). Subsequent alignment to the reference genome showed an average mapping rate of 96.5%, with 89% of paired reads uniquely aligned, ensuring efficient downstream differential expression analysis.

### 3.4. Identification of DEGs in Muscle and Liver Samples of Deccani and Nellore Sheep Breeds

**Figure 3:**
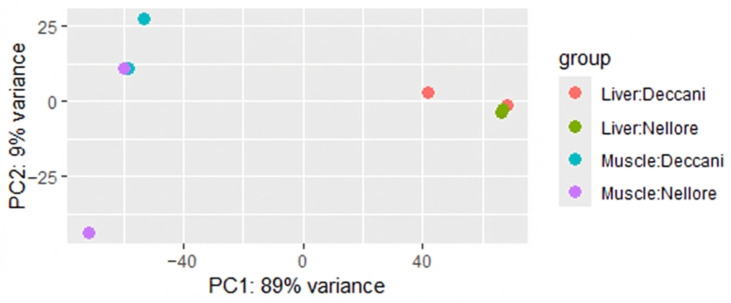
Principal component analysis (PCA) of muscle and liver samples from Deccani and Nellore sheep breeds. PC1 accounts for tissue variation, and PC2 accounts for breed-specific variation.

There are 30,722 read counts, of which 30,319 have nonzero total read counts and are used for preprocessing and differential gene expression analysis. The principal component analysis (PCA) of gene expressions in muscle and liver samples of Deccani and Nellore breeds is shown in Figure 3. The first Principal Component Analysis (PCA1) explains 89% of the total variance, clearly separating muscle from liver samples. The second Principal Component Analysis (PCA2) accounts for 9% of the total variation in gene expression between muscle samples of the Nellore and Deccani breeds.

**Figure 4:**
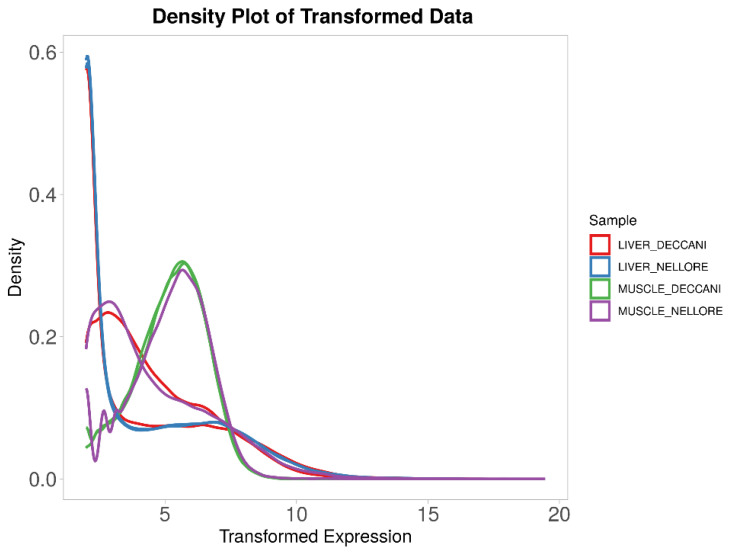
Fragments per kilobase of exon per million mapped fragments (FPKM) density distribution of muscle and liver samples for Deccani liver (red line), Nellore liver (blue line), Deccani muscle (green line), and Nellore muscle (purple line).

**Figure 5:**
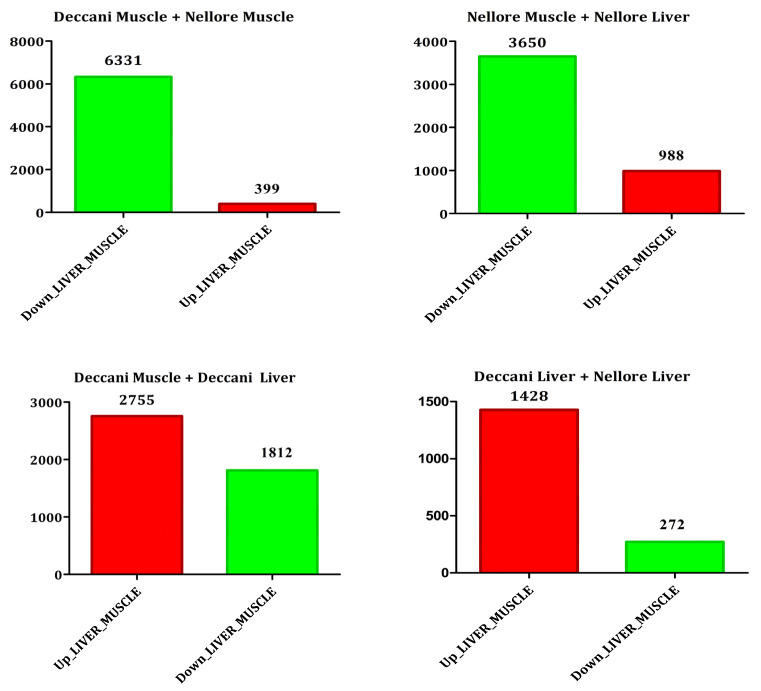
UpSetR plot representing the number of up- and down-regulated genes in all the comparisons.

**Figure 6:**
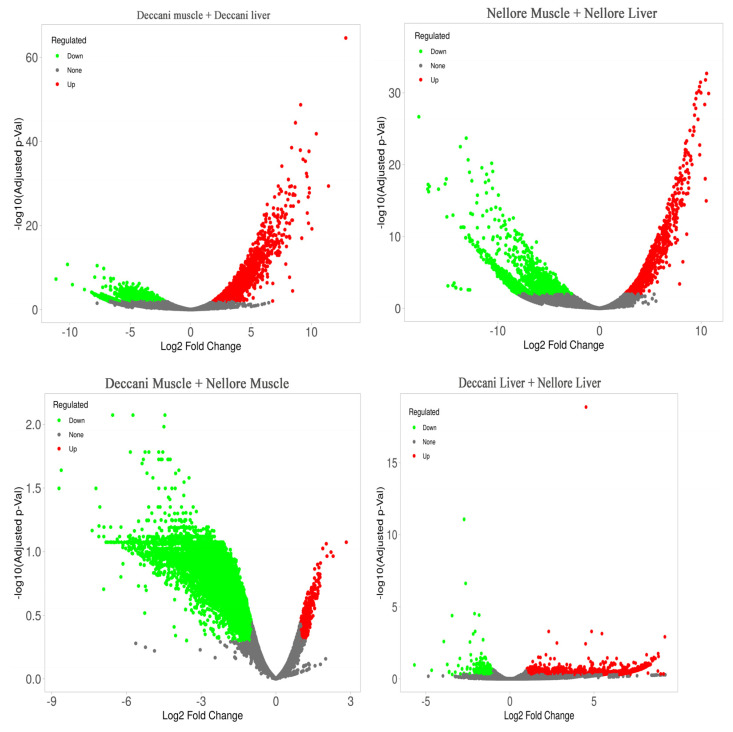
Volcano plot depicting differentially expressed genes in Deccani muscle + Deccani liver, Nellore Muscle + Nellore Liver, Deccani Muscle + Nellore Muscle, Deccani Liver + Nellore Liver. Red dots represent genes expressed at higher levels, while blue dots represent genes with higher expression levels. Grey dots indicate no significant change. The *Y*-axis denotes −log10 (Adjusted *p* values), while the *X*-axis shows log2 fold change values. The volcano plot was generated using R software.

The muscle and liver samples from all treatment groups showed a similar skewed distribution of FPKM-transformed expression density, with approximately 40–60% of genes being lowly expressed (Figure 4). For differential expression analysis, the samples were grouped as Muscle and Liver. The expression profile of differentially expressed genes across the samples is presented in bar plots (Figure 5). When comparing muscle and liver within Deccani breed, a higher number of genes were up-regulated (2755 genes) than down-regulated (1812 genes). In contrast, the Nellore breed showed a predominance of down-regulated genes (3650 genes) compared to up-regulated genes (988 genes) between muscle and liver. In muscle tissue, Deccani sheep exhibited a substantially larger number of down-regulated (6331 genes) relative to Nellore, whereas only 399 genes were up-regulated. Conversely, in liver tissue, Deccani sheep showed a high number of up-regulated genes (1428 genes) compared to down-regulated genes (272 genes). Volcano plots were generated to visualise the differentially expressed genes between the groups (Figure 6).

**Figure 7:**
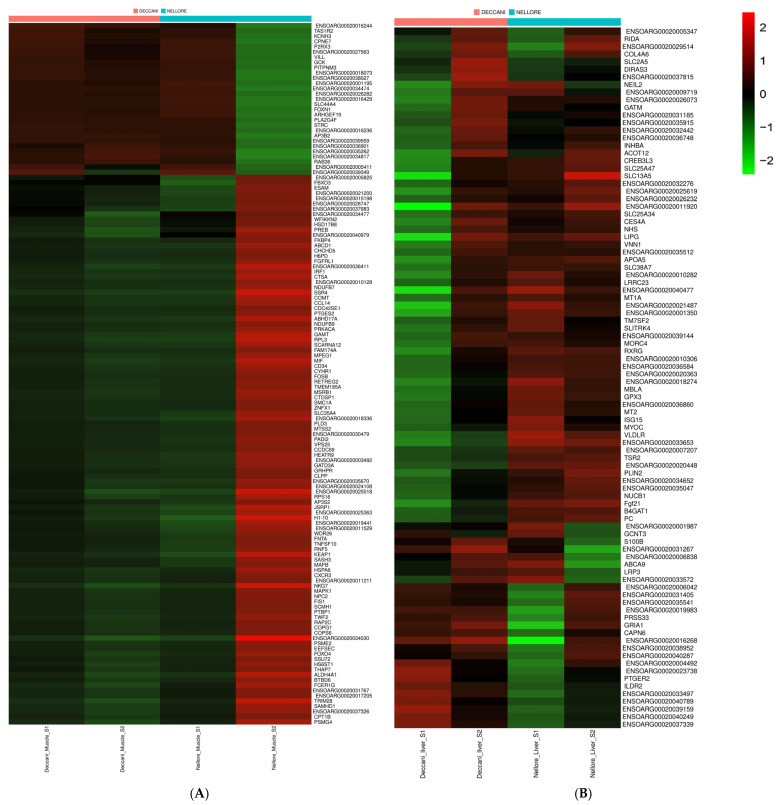
A heatmap of the DEGs related to growth, development, and meat quality in (**A**) Deccani Muscle Vs. Nellore Muscle and (**B**) Deccani Liver Vs. Nellore Liver. The DEGs were detected by DESeq2 (v1.30.1) with |log2(fold change)| ≥ 2. The green-coloured lines identify a lower level of gene expression, while the red-coloured lines indicate a higher level of gene expression.

A heatmap was constructed using these 100 DEGs (Figure 7). A total of upregulated and downregulated DEGs showed a significant difference between the Deccani × Nellore sheep breeds. However, some DEGs did not show significant differences when visualised via the heatmap. This gene expression profiling provides valuable genetic markers and biological insights that can support future molecular breeding programmes aimed at enhancing economically significant traits in indigenous sheep populations.

In Deccani sheep, upregulated genes were largely related to lipid metabolism, glycolysis, ion transport, and sensory-metabolic signalling. PLA2G4F (Phospholipase A2 Group IV F), and GCK (Glucokinase), were elevated. Other upregulated genes in Deccani included PITPNM3 (PITPNM Family Member 3), KCNH3 (Potassium Voltage-Gated Channel Subfamily H Member 3).

Conversely, in Nellore sheep, genes linked to muscle development, mitochondrial activity, thermotolerance, and immune function were prominently upregulated. These included WFIKKN2 (growth and differentiation factor associated serum protein-1, also called GASP1) [32], and NDUFB7 (NADH: Ubiquinone Oxidoreductase Subunit B7. TAS1R2 (Taste receptor type 1 member 2), a gene traditionally known for sweet taste signalling, was downregulated in Nellore muscle. Additionally, FKBP4 (FK506-binding protein 4), and H6PD (Hexose-6-Phosphate Dehydrogenase), and IRF1 (Interferon regulatory factor 1), showed higher expression.

### 3.5. Functional Enrichment Analysis of DEGs

**Figure 8:**
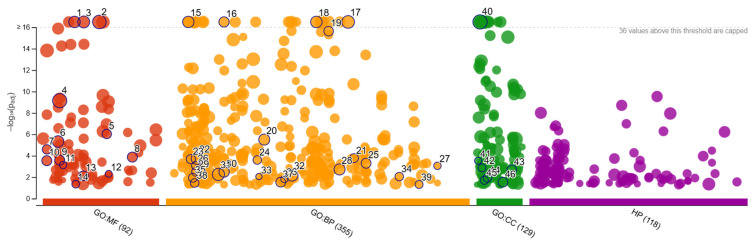
Functional enrichment of DEGs in muscle and liver samples using g: Profiler. The numbers displayed on individual data points in the figure correspond to the indexed enriched GO terms listed in Figure A1. Red—Molecular Function (MF), Orange—(Biological Processes), Green—Cellular Function, Purple—Human Phenotype Ontology (HP) database predicted for Deccani and Nellore sheep breeds.

**Figure A1:**
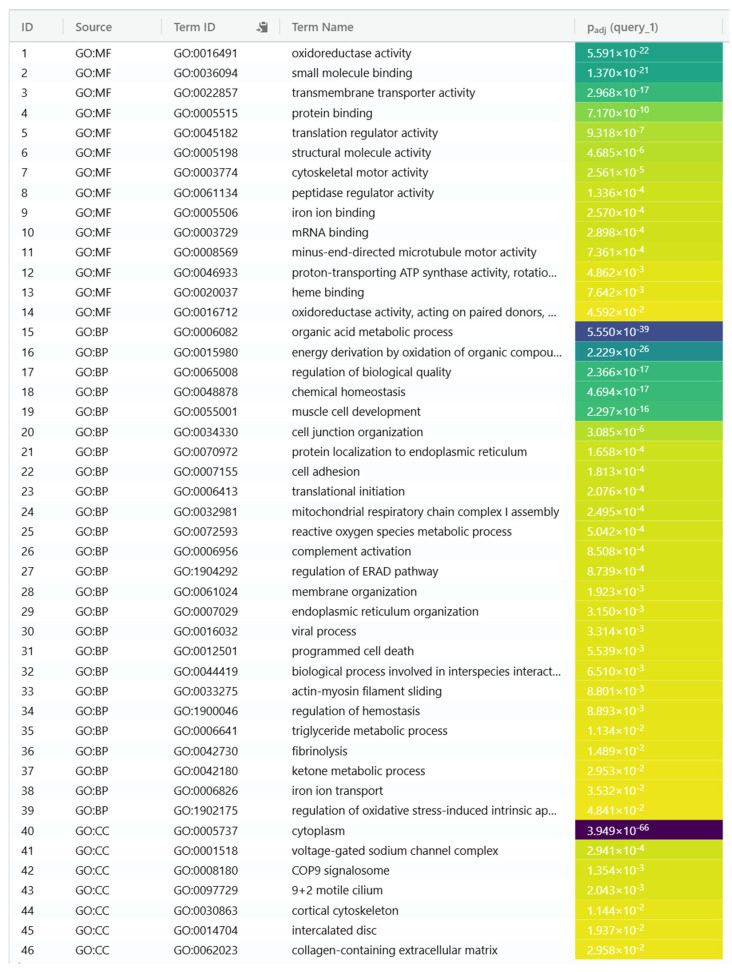
Top 46 significantly enriched terms for the DEGs by Gene Ontology database, which were shown in Figure 7A.

A functional enrichment analysis of DEGs associated with IMF deposition and fatty acid metabolism in Deccani and Nellore sheep breeds revealed significant enrichment across 46 Gene Ontology (GO) terms under Molecular Function (MF), Biological Process (BP), and Cellular Component (CC) categories (Figure 8 and Figure A1).

In the Molecular Function category, oxidoreductase activity (GO:0016491) was the most highly enriched term, involving 228 genes. Among these, genes such as ACADL (Acyl-CoA dehydrogenase, long-chain), CYP4A11 (Cytochrome P450 family 4 subfamily A member 11), and DHCR24 (24-dehydrocholesterol reductase) were particularly notable. Additionally, the terms small molecule binding (GO:0036094) and ion binding (GO:0043167) were significantly enriched, encompassing 974 and 932 genes, respectively. Key genes under these categories included FABP4 (Fatty Acid Binding Protein 4) and APOA1 (Apolipoprotein A1). Furthermore, transmembrane transporter activity (GO:0022857), with 296 genes, included important genes such as SLC27A1 (Fatty acid transporter protein 1) and ABCA1 (ATP-binding cassette transporter A1).

The Biological Process enrichment revealed that the fatty acid metabolic process (GO:0006631), lipid catabolic process (GO:0016042), and regulation of lipid metabolic process (GO:0019216) were among the most significantly represented pathways. Genes like ACSL1 (Acyl-CoA synthetase long-chain family member 1), ACOX1 (Acyl-CoA oxidase 1), and CPT1A (Carnitine palmitoyltransferase 1A) featured prominently in these categories. Additionally, LPL (Lipoprotein lipase) and PLIN1 (Perilipin 1) were enriched in lipid storage and triglyceride metabolism. The enrichment of oxidation-reduction processes (GO:0055114), with genes like NQO1 (NAD(P)H Quinone Dehydrogenase 1) and GPX3 (Glutathione Peroxidase 3), suggests a link between lipid metabolism and oxidative stress management within muscle tissues.

In the Cellular Component category, the mitochondrial matrix (GO:0005759) was the most significantly enriched location, with 69 genes including CPT2 (Carnitine palmitoyltransferase 2), HADHA (Hydroxyacyl-CoA dehydrogenase trifunctional multienzyme complex subunit alpha), and ACADVL (Very long-chain specific acyl-CoA dehydrogenase). The peroxisome (GO:0005777), represented by 24 genes such as ACOX1 and PEX5 (Peroxisomal biogenesis factor 5), was also significantly enriched. Moreover, the lipid droplet (GO:0005811), associated with 13 genes including PLIN1, PLIN2 (Perilipin 2), and DGAT2 (Diacylglycerol O-Acyltransferase 2), was enriched.

This comprehensive enrichment analysis highlights a tightly regulated network of genes and pathways involved in fatty acid metabolism, lipid transport, and intracellular storage mechanisms. The coordinated action of genes such as ACADL, CPT1A, FABP4, LPL, PLIN1, and DGAT2 points to a complex balance between lipid deposition and utilisation within intramuscular fat tissues, directly influencing the marbling characteristics, tenderness, and nutritional value of meat in Deccani and Nellore sheep breeds. The findings emphasise breed-specific variations in subcellular lipid metabolism and provide valuable molecular insights into fat deposition traits in livestock.

## 4. Discussion

A comprehensive transcriptomic comparison between Deccani and Nellore sheep breeds using RNA-Seq-based transcriptomic profiling lays the foundation for genetic improvement initiatives. The comparative transcriptomic analysis of Deccani and Nellore sheep reveals unique gene expression profiles that reflect their divergent physiological adaptations, muscle development strategies, and fat metabolism pathways, all of which contribute to differences in meat quality, carcass characteristics, and environmental resilience. These differences underscore the selection and evolutionary forces acting upon each breed and provide valuable insights for genetic studies enhancing both performance and meat quality traits.

The comparison of carcass characteristics between Nellore and Deccani sheep revealed distinct breed-specific differences reflecting their divergent production potentials. Nellore sheep exhibited higher Slaughter Body Weight, HCW, CCW, HCY, and CCY, indicating superior growth performance and lean meat accretion [33]. No significant difference was observed in cooling loss between breeds, suggesting that both breeds have good water retention during chilling. In contrast, Deccani sheep showed relatively lower Carcass weights and yields, consistent with their smaller body frame [34].

Whereas the histological evaluation of the longissimus dorsi muscle further supported these variations. Nellore sheep showed relatively larger myofibrillar cross-sectional area, which is commonly associated with muscle hypertrophy and lean carcass composition. While, the Deccani breed showed significantly higher IMF deposition which is commonly associated with improved meat tenderness and flavour perception.

The transcriptomic profile of Nellore sheep revealed the upregulation of WFIKKN2 and FGFRL1 genes, which have been previously reported to influence muscle fibre regulation and growth-related pathways in sheep and other livestock species [35,36,37,38,39,40,41]. The upregulation of genes such as WFIKKN2 and FGFRL1 in Nellore provides a molecular basis for the observed enhanced muscle growth capacity and improved carcass characteristics (live weight, HCW, CCW, HCY, and CCY along with increased myofibrillar cross-sectional area and lower IMF content). WFIKKN2 (muscle cell development GO:0055001), a known inhibitor of myostatin and GDF11 (Growth Differentiation Factor 11), modulates the TGF-β signalling pathway [42] promoting muscle fibre hypertrophy by the suppression of negative regulators of muscle growth. This molecular function has been validated in transgenic mouse models, where overexpression of WFIKKN2 significantly increases muscle mass and alters muscle fibre composition [32,43]. In livestock, particularly in sheep, the study by Wang et al. [44] provided the first evidence of WFIKKN2 polymorphism influencing carcass muscle traits, notably in a gender-dependent manner. A recent study by Kong et al. [36] found that WFIKKN2 was linked to muscle growth and development in Southdown × Hu F1 sheep, a finding consistent with its upregulation in the current study.

The upregulation of FGFRL1 observed in Nellore sheep provides a plausible molecular explanation for the larger myofibrillar cross-sectional area and leaner carcass composition in this breed (as shown in Figure 1A,B and Figure 2A and Table 1). Previous research in sheep and cattle is substantially supported by our findings. FGFRL1 has been identified as a gene potentially linked to muscle growth traits such as fibre differentiation, cell adhesion (GO:0007155), and bone formation, and was identified as a candidate gene linked to muscle growth traits [45,46]. The presence of these growth-favouring genes suggests a transcriptional landscape that prioritises muscle growth, consistent with Nellore’s reputation for rapid postnatal growth and lean carcass characteristics. TAS1R2’s downregulation in Nellore sheep suggests its potential role in mitochondrial activity, muscle mass, and fibre structure.

Further experimental data have uncovered its involvement in skeletal muscle physiology. In particular, muscle-specific knockout of TAS1R2 in mice resulted in increased lean mass, larger myofiber cross-sectional area, and enhanced mitochondrial activity [47]. This may align closely with our findings in Nellore sheep, where downregulation of TAS1R2 was observed alongside larger muscle fibres, leaner carcasses and improved carcass traits. These parallels support the hypothesis that TAS11R2 may act as a negative regulator of muscle growth, and its suppression could contribute to the breed’s superior muscle phenotype. Although such functional roles have not yet been fully validated in ruminants, our results lend support to the hypothesis that TAS1R2 downregulation in Nellore facilitates muscle growth by improving the bioenergetics of the muscle.

The anabolic profile exhibited is further supported by the upregulation of genes related to oxidoreductase activity, such as ACADL, CYP4A11, DHCR24, and NDUFB7, which underscores the importance of redox regulation in lipid catabolism and mitochondrial energy metabolism. These enzymes may play a key role in oxidising fatty acids for ATP generation, significantly contributing to energy metabolism and fat mobilisation in muscle tissues [48,49]. The presence of these DEGs predominantly in the Nellore muscle further emphasises this breed’s adaptation towards rapid lipid mobilisation and leaner carcass composition. Further, FKBP4 encodes a heat shock protein-binding immunophilin that plays a critical role in protein folding, cellular protection under thermal stress, and steroid receptor signalling. Our results align with recent findings by Maxman et al. [50], who identified FKBP4 within selection signatures associated with adaptation and heat tolerance in South African indigenous cattle. The upregulation of FKBP4 in Nellore sheep provides molecular evidence of the breed’s potential for enhanced cellular stress response and thermotolerance capacity. FKBP4’s role in heat stress pathways, including interactions with HSP70 and HSP90, makes it a relevant candidate gene for thermotolerance in tropical production systems [51]. Furthermore, the upregulation of IRF1 in Nellore sheep may contribute to the breed’s superior ability for immune regulation and stress adaptation. IRF1 is a transcription factor pivotal in mediating immune response, predominantly in the activation of interferon-stimulated genes. According to Wen et al. [52] IRF1 is involved in resistance to viral infections in goats and in enhancing innate immunity via IRF3 activation. These findings are consistent with our results, supporting the hypothesis that this breed is more immunologically prepared [33].

In the Biological Process (BP) category, significant mechanisms such as fatty acid metabolic process (GO:0006631) and lipid catabolic process (GO:0016042) were markedly enhanced. Genes like ACSL1, ACOX1, and CPT1A were prominent in these pathways. These genes are fundamental in facilitating FA activation, transport, and oxidation, vital for balancing lipid storage and energy requirements [53,54]. The coordinated upregulation of these genes in Deccani sheep muscle provides molecular support for a higher capacity for IMF deposition, a finding consistent with their superior meat tenderness and flavour, traits that are previously linked with marbled meat [4].

Moreover, genes associated with lipid droplet formation and triglyceride metabolism, including DGAT2 (Diacylglycerol O-Acyltransferase 2), PLIN1 (Perilipin 1), and PLIN2 (Perilipin 2), reflecting their role in lipid droplet formation and regulation of lipolysis within intramuscular fat depots were differentially expressed in the Deccani breed, a finding consistent with their established regulatory role in neutral lipid storage within muscle fibres [55,56]. These findings are consistent with recent reports demonstrating the involvement of PLIN proteins in adipocyte lipid storage, turnover, and protection of lipid droplets from lipolysis, directly affecting IMF content [54].

The enrichment of cellular compartments identified in the present study, such as the mitochondrial matrix (GO:0005759) and peroxisomes (GO:0005777), lends credence to the hypothesis that fat metabolism in Deccani and Nellore sheep is tightly regulated at the organelle level. Genes including CPT2, HADHA, and ACADVL were primarily localised within the mitochondria, highlighting their role in fatty acid β-oxidation and ATP production [57]. Likewise, ACOX1 and PEX5 are crucial peroxisomal genes regulating very long-chain fatty acid catabolism, and indicate peroxisomal critical role in the initial breakdown of long-chain and branched-chain fatty acids before their transfer to mitochondria for complete oxidation [58], indicating an additional layer of lipid regulation specific to Deccani sheep muscle.

Regulatory genes such as PPARG and LEPR (Leptin Receptor), which modulate adipocyte differentiation, lipid storage, and energy homeostasis, also exhibited significant variations in their expression. Their upregulation in Deccani sheep is consistent with the breed’s higher IMF and metabolic resilience, highlighting the intricate interplay between genetic regulation, fat metabolism, and phenotypic traits in indigenous sheep [34]. PPARG’s central role in adipogenesis has been widely documented in both small ruminants and cattle [53]. Its involvement in Deccani sheep provides molecular evidence for valuable opportunities for marker-assisted selection to enhance meat quality.

Comparative transcriptomic evidence indicates that Nellore sheep exhibit a gene expression profile favouring muscle development, rapid lipid mobilisation and energy-efficient growth, reflected in their lean carcass traits and faster growth rates. In contrast, Deccani sheep demonstrated enhanced expression of genes related to lipid storage, triglyceride synthesis, and mitochondrial oxidation, consistent with their dual-purpose of meat-wool phenotype and adaptation to semi-arid climates. Although the sample size was limited, this work provides an initial framework for understanding gene expression dynamics under an extensive feeding (grazing) system. This study not only enhances our understanding of genotype–phenotype relationships in the selected Indian sheep but also lays the foundation for genomic selection strategies aimed at improving meat production and quality. The identified genes and enriched pathways can serve as valuable resources for future functional genomics, validation studies, and marker-assisted breeding programmes aimed at enhancing the productivity, quality, and adaptability of India’s indigenous sheep genetic resources. Future investigations incorporating larger cohorts and qPCR-based validation are needed to substantiate these findings and facilitate the use of the identified genes as potential molecular markers.

## 5. Conclusions

This study constitutes one of the first comprehensive investigations integrating transcriptomic and functional enrichment analyses exploring muscle development, intramuscular fat content, and fatty acid metabolism in Deccani and Nellore sheep breeds. Through RNA-Seq and differential gene expression analysis, it is evident that Nellore sheep exhibited a gene expression signature favouring rapid muscle development, oxidative metabolism, lean carcass composition, and climate resilience (upregulation of WFIKKN2, FKBP4, IRF1 and downregulation of TAS1R2), which are beneficial for commercial meat production. In contrast, Deccani sheep demonstrate a genetic architecture that supports IMF deposition and lipid storage (upregulation of PLA2G4F, ACSL1, ACOX1, CPT1A, and PLIN1), which are associated with tender and flavour-rich meat. These findings provide novel genetic markers and biological insights into the regulation of muscle development, thermotolerance, immunity, and IMF and fat metabolism in indigenous Indian sheep breeds. The identified DEGs can serve as marker genes in marker-assisted selection (MAS) programmes for improving indigenous breeds, in enhancing lean growth, thermotolerance, marbling, and meat quality traits. A key limitation of this study is the relatively small sample size; nevertheless, this work provides an initial framework for understanding gene expression dynamics in Nellore and Deccani sheep breeds. However, to successfully integrate this information into MAS programmes, further studies on the functional validation of these key genes and integration with Genome-Wide Association Studies (GWAS) across larger populations are warranted.

## Figures and Tables

**Table 1:** Means of carcass characteristics and age of Nellore and Deccani sheep breeds.

Sl. No.	Slaughter Characteristics and Age of the Animal	Deccani (*n* = 4)	Nellore (*n* = 4)	*p*-Value
1.	Live Weight (Slaughter Body Weight)	19.50 ± 0.96	23.96 ± 0.89	0.0146
2.	Hot Carcass Weight—HCW (Kg)	9.00 ± 0.41	11.89 ± 0.45	0.0032
3.	Cold Carcass Weight—CCW (Kg)	8.38 ± 0.50	11.21 ± 0.38	0.0048
4.	Hot Carcass Yield (HCY) (%)	46.20 ± 0.81	49.61 ± 0.18	0.0215
5.	Cold Carcass yield (CCY) (%)	42.94 ± 1.08	46.81 ± 0.29	0.0330
6.	Cooling Loss (%)	7.06 ± 1.30	5.63 ± 0.71	0.4010
7.	Age (in months)	10.5 ± 1.1	10.8 ± 1.2	0.5210

## Data Availability

The data presented in this study are openly available in NCBI Sequence Read Archive (SRA) at https://www.ncbi.nlm.nih.gov/search/all/?term=PRJNA1293839, accessed on 28 December 2025.

## Supplementary Materials

The following supporting information can be downloaded at: https://www.mdpi.com/article/10.3390/ani16030452/s1, Table S1: Summary of sequencing results of Longissimus dorsi muscle and liver samples from Deccani and Nellore sheep breeds; Table S2: Gene Ontology (GO) functional enrichment analysis results; Figure S1: Mean Phred quality scores across read positions showing consistently high base quality (Q ≥ 30); Figure S2: Distribution of per-sequence quality phred quality scores indicating oveall high-quality reads (Q ≥ 30).
