# Supplementary material for: Transcriptomic Exploration of Muscle Development and Fat Deposition Trait Diversity in Selected Indian Sheep Breeds: Implications for Meat Quality and Yield
Source: Animals (Basel). 2026 Feb 1;16(3):452. doi: 10.3390/ani16030452 (PMC12896845; doi:10.3390/ani16030452)
Supplement: Supplementary file 1 [file animals-16-00452-s001.zip › Supplementary Data.pdf]

Table S2: Summary of sequencing results of Longissimus dorsi muscle and liver samples from Deccani and Nellore sheep breeds

Samples	Samples Type	Raw reads	Clean reads	Unique Reads	Mapped Reads	Total mapping %	Read length	Q30%	GC Content
Deccani	Muscle-R1	57.848548	57.271134	22616344	28924274	82.15%	159	92.51	47.2
Deccani	Muscle-R2	57.848548	57.271134	20744689					
Deccani	Muscle-R1	59.351242	58.284246	21625857	29675621	74.79%	159	90.7	45.8
Deccani	Muscle-R2	59.351242	58.284246	21535548					
Deccani	Liver-R1	49.067126	48.357484	13279850	24533563	85.32%	159	92.9	45.3
Deccani	Liver-R2	49.067126	48.357484	13229525					
Deccani	Liver-R1	29.204666	28.626866	5066390	17669315	63.48%	159	93.7	44.2
Deccani	Liver-R2	29.204666	28.626866	5044917					
Nellore	Muscle-R1	56.042284	54.325274	8938138	28021142	81.61%	159	91.6	48.9
Nellore	Muscle-R2	56.042284	54.325274	8916115					
Nellore	Muscle-R1	24.419476	24.068888	10365163	16098921	83.63%	159	92.4	47.4
Nellore	Muscle-R2	24.419476	24.068888	10208955					
Nellore	Liver-R1	42.238712	41.499592	7690576	23933226	85.55%	159	94.1	47.4
Nellore	Liver-R2	42.238712	41.499592	7650210					
Nellore	Liver-R1	49.002652	48.155848	6467574	27593564	85.90%	159	93.6	46.6
Nellore	Liver-R2	49.002652	48.155848	6471772					

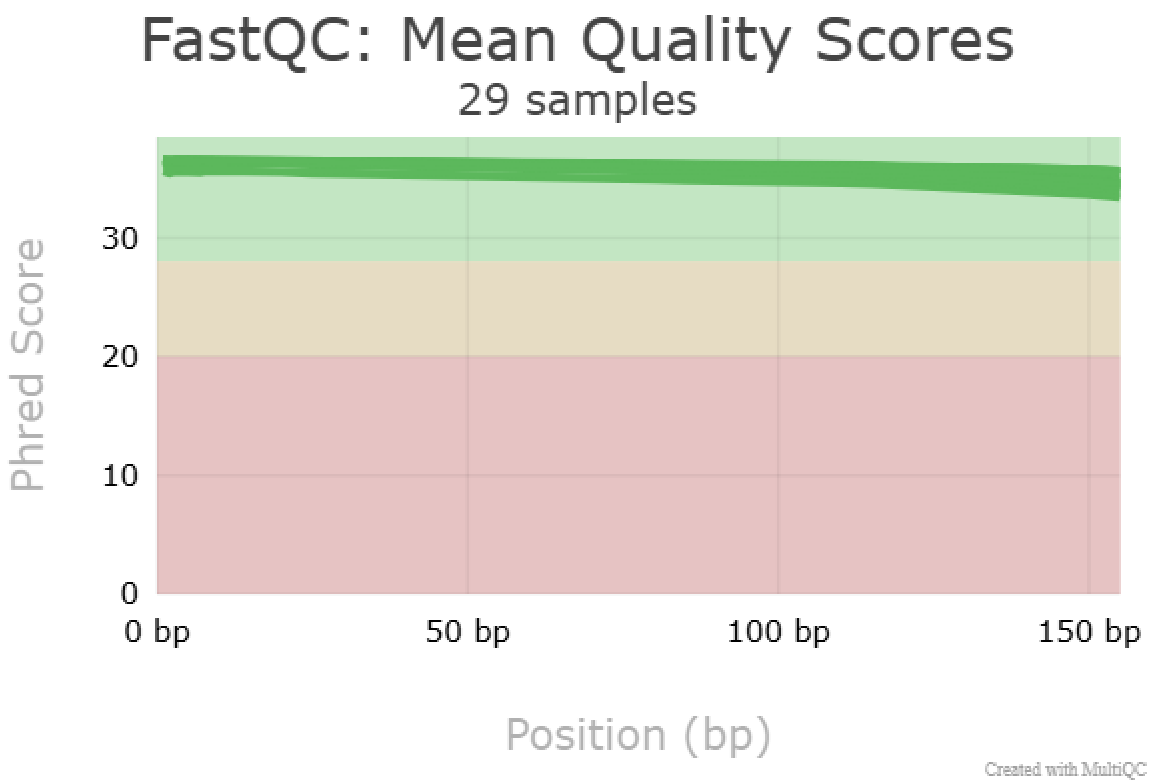

Figure S1: Mean Phred quality scores across read positions showing consistently high base quality (Q ≥ 30)

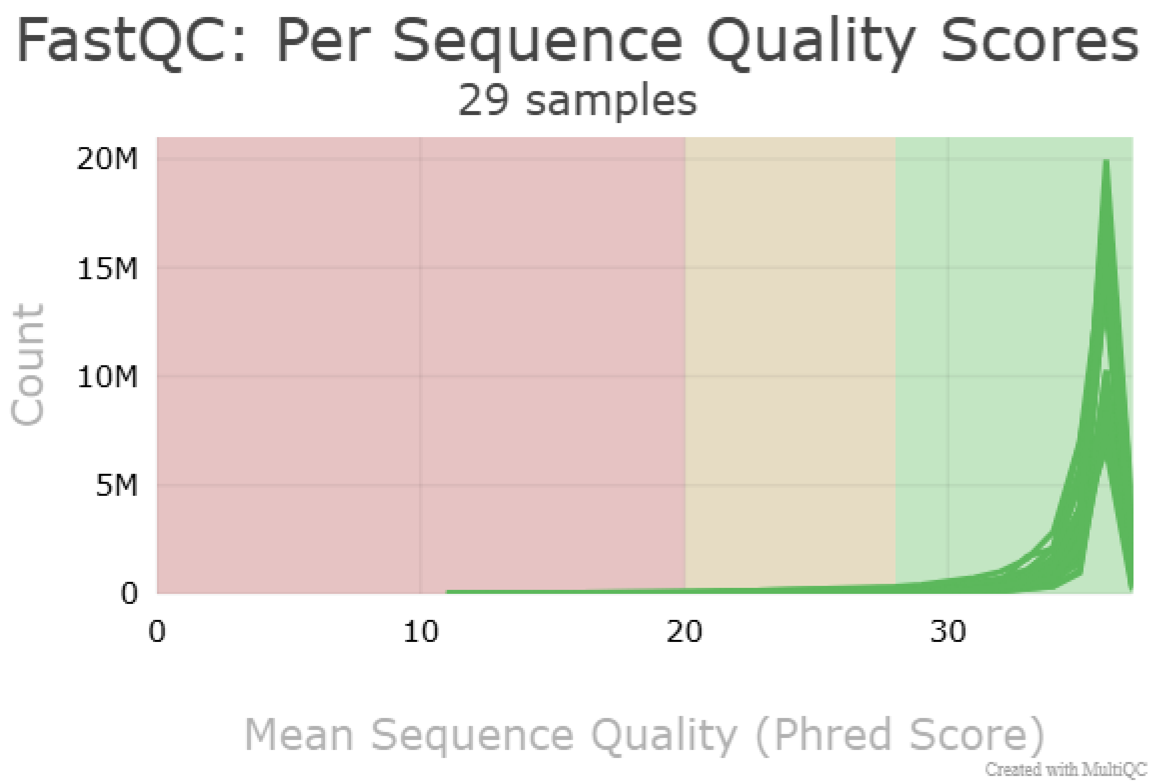

Figure S2: Distribution of per-sequence quality phred quality scores indicating oveall high-quality reads (Q ≥ 30)
